# Infection with *Cryptosporidium parvum* Affects Secondary Sexual Characteristics of Male Mice by Altering the Pheromone Content in Preputial Gland

**DOI:** 10.3390/ani13040756

**Published:** 2023-02-19

**Authors:** Gaojian Li, Tao Zhang, Bin Hu, Shuyi Han, Chen Xiang, Guohui Yuan, Hongxuan He

**Affiliations:** 1 National Research Center for Wildlife-Borne Diseases, Institute of Zoology, Chinese Academy of Sciences, Beijing 100101, China; 2 University of Chinese Academy of Sciences, Beijing 100101, China

**Keywords:** *Cryptosporidium parvum*, parasitic infection, pheromone changes, secondary sexual characteristics, ICR/CD-1 male mice

## Abstract

**Simple Summary:**

During the breeding process of mice, the females can evaluate the health status of males and choose the suitable mates through the smell of urine. Under the natural conditions, parasitic infections in small mammals are very common, and parasitic infections can affect the health status of hosts. However, the impact of parasitic infection on mammalian reproductive capacity has not been fully studied. In this study, we used the ICR/CD-1 mouse model to explore the relationship between parasitic infection and pheromone content; *Cryptosporidium parvum* was used as the infectious agent. This study revealed that the urine attractiveness of the ICR/CD-1 male mice was decreased after parasitic infection when compared with the uninfected group. Both the gene expression level and protein concentration of MUP in liver and urine were down-regulated after parasitic infection. The results of GC-MS analysis indicated that the contents of different pheromones were significantly reduced after parasitic infection. In summary, this study revealed that infection with *C. parvum* affects the secondary sexual characteristics of male ICR/CD-1 mice and alters the pheromone contents in the foreskin gland.

**Abstract:**

The olfactory acuity of female mice allows them to discriminate the urinary odors of males. Parasitic infection can reduce the odor attractiveness of male mice to females and result in female aversion or avoidance responses in odor selection. However, the chemical signaling changes in the pheromone contents produced by the foreskin gland were not fully revealed after parasitic infection. *Cryptosporidium parvum* (*C. parvum*) is a common zoonotic intestinal parasite and has a wide range of hosts, including human, domestic animals, and wild animals. In this study, we immunosuppressed ICR/CD-1 male mice by dexamethasone sodium phosphate treatment. After *C. parvum* infection, physiological indexes such as body weight and organ weight were significantly decreased. Furthermore, the gene expression level of MUP (major urinary protein) in liver and urine were significantly down-regulated, which could be the reason for the decrease in urine attractiveness to females. GC-MS was performed to analyze the changes in the pheromone produced by the preputial gland before and after parasitic infection, and the results indicated that the levels of different pheromones were significantly reduced after parasitic infection. In summary, this study reveals that *C. parvum* infection damages the secondary sexual characteristics of male ICR/CD-1 male mice and decreases the pheromone content produced by the foreskin gland.

## 1. Introduction

The health status of male mice can influence the mate choice of females, and female mice prefer parasite-free or parasite-resistant males [1]. The invasion of infectious agents changed the health status of male mice, and the females could discriminate the uninfected male mice [2]. In the process of choosing a partner, chemical communication can provide effective information to reflect the personal health and infection status of a mate. It should be noted that the odor of male mouse urine has a profound effect on *Mus musculus* partner choice [3].

Urine is an important chemical signal source in finding a mate, urine contains a class of lipocalin named the major urinary proteins (MUPs). MUPs play a crucial role in chemical communication as they can bind, concentrate, and slowly release volatile pheromones [4]. Furthermore, MUPs are important signals of the competitive abilities of successfully territorial males [5]. The expression of MUPs is not stable [6,7,8]; the expenditure in sexual signals used in mate choice can be influenced by the health status of male mice [9]. Both the concentration and fractional composition of MUPs shape the individuality and attractiveness of male scent marks [10].

*C. parvum* is a common pathogen that causes cryptosporidiosis; it can propagate in many animal species including mammals, marsupials, reptiles, birds, and fish [11]. *C. parvum* is an obligately intracellular parasite and it can infect the epithelial cells in the luminal surfaces of the digestive and respiratory tracts. This parasite can be excreted and exist in the environment as a dormant resistant oocyst [12]. One study reported that infection with *Toxoplasma gondii* could block the innate aversion of rats for cat urine, instead producing an attraction to the pheromone [13]. However, the pheromone changes caused by *C. parvum* have hardly been studied.

In this study, we investigate the changes in pheromone contents caused by *C. parvum* infection in ICR/CD-1 male mice. Parasitic infection down-regulated the expression level of MUPs in live and urine, which could reduce the pheromone content in the foreskin gland and further decrease the attractiveness of male mice urine to females. In summary, this study revealed that acute infection with *C. parvum* could down-regulate the pheromone content in the foreskin gland and affect the secondary sexual characteristics of ICR/CD-1 male mice.

## 2. Materials and Methods

### 2.1. Animals 

ICR/CD-1 mice (8–10 weeks old) were purchased from Weitong-Lihua Experimental Animal Company, Beijing, China. Both the males and females were kept in plastic cages, and the temperature was set to 22 ± 0.5 °C. Males were individually kept and females were kept in groups of three; food and water were provided freely. All mice were virgin and in estrus, males were scrotal, and females had perforated vaginas. Before the experiment, the mice were adapted to this environment for 2 weeks. The protocols for animal studies were approved by the Committee on the Ethics of Animal Experiments of the Institute of Zoology, Chinese Academy of Sciences (Approval number: IOZ20211010-08). Humane treatment was administered according to the 3R principle during the entire investigation [14].

### 2.2. Oocysts Isolation and Culture Maintenance

The *C. parvum* was a gift from Dr. Longxian Zhang (Department of Animal Science, Henan Agricultural University, Henan, China), and the parasite continued to be propagated in newborn calves via collection of feces on the third day after infection [15]. Oocysts were purified by sucrose density gradient centrifugation as described previously [16]. After purification, the oocysts were maintained for a short time according to a previously published method until the isolated oocysts were sufficient for animal experiment [17]. In brief, the oocysts were kept at room temperature in 100 mL of RPMI-1640 medium (pH 7.4) supplemented with 0.03 g L-glutamine, 0.3 g sodium bicarbonate, 0.02 g bovine bile, 0.1 g glucose, 25 mg folic acid, 100 mg 4-aminobenzoic acid, 50 mg calcium pantothenate, 875 mg ascorbic acid, 1% FCS, 0.36 g HEPES buffer, 10,000 U penicillin G, and 0.01 g streptomycin. The maintenance incubation time for the oocysts obtained from a single isolation was no longer than three days.

### 2.3. Mouse Immunosuppression

Dexamethasone had been reported as an effective drug to induce immunosuppression. In this study, 20 male ICR/CD-1 mice were randomly divided into two groups (Dex group and control group) and 10 mice per group, the Dex group, were intraperitoneally injected with 200 μL of dexamethasone sodium phosphate (5 mg/mL) dissolved in sterile water (Solarbio, Beijing, China) every other day [18,19]. For the control group, the mice received 500 μL of 0.9 % normal saline in the same manner. The immunosuppression experiment lasted for 14 days, and the mice underwent seven injections with either dexamethasone sodium phosphate or 0.9% normal saline.

### 2.4. Lymphocyte Proliferation Assay

To evaluate the treatment effect of dexamethasone sodium phosphate, a lymphocyte proliferation assay was performed by a previously published method [20]. In brief, the splenic lymphocytes were separated by a lymphocyte separation medium (Dakewe Biotech Co., Ltd., Shenzhen, China). The lymphocytes were seeded into plates at 5 × 10^5^ cells/well, and each splenic lymphocyte sample was plated in triplicate. Two micrograms of Concanavalin A (Sigma-Aldrich, Burlington, MA, USA) dissolved in 100 μL of RPMI-1640 medium was added as a positive control, and 100 μL of RPMI-1640 medium was added as a negative control. Subsequently, the lymphocytes were cultured at 37 °C with 5% CO_2_ for 36 h, then the cell proliferative activity was measured by standard MTT assay as described previously [21]. The optical density (OD) was determined at wavelengths of 570 nm and 630 nm; the stimulation index (SI) was calculated with the following formula, SI = positive control (Concanavalin A) mean OD_570_ − OD_630_ / negative control (RPMI-1640) mean OD_570_ − OD_630_.

### 2.5. Flow Cytometry

Peripheral blood samples were collected from tail veins and transferred to heparin tubes, then the peripheral blood was treated with red blood cell lysis buffer (Solarbio, Beijing, China) and the white blood cells were collected by centrifugation at 450× *g* for 10 min at 4 °C. The white blood cells were washed again with red blood cell lysis buffer and collected by centrifugation. Then, the white blood cells were suspended in D-hanks Balanced Salt Solution (Solarbio, Beijing, China) at 10^6^ cells/mL, and 30 μL of cell suspension was treated with FITC-labeled mouse monoclonal anti-CD4 (Abcam, China) or PE-labeled mouse monoclonal anti-CD8 (Abcam, Beijing, China) for 30 min at 4 °C. Then, the cells were washed with D-hanks Balanced Salt Solution and the percentage of the CD4^+^ and CD8^+^ cell subpopulations was determined by flow cytometry (MoFlo XDP, Beckman, CA, USA).

### 2.6. C. parvum Infection

Infection with *C. parvum* was performed according to previous published methods [22,23]. In brief, 20 ICR/CD-1 male mice were divided into two groups (infection group and control group) and 10 mice per group. Fourteen days before parasitic infection, the mice were immunosuppressed by dosed dexamethasone sodium phosphate at 1.5 mg per 100 mL in the drinking water. Then, the infection group was intragastrically inoculated with 1 × 10^5^ *C. parvum* oocyst larvae for each mouse, suspended in 0.1 mL of PBS. After parasitic infection, both the control and infection groups were kept for another 14 days to eliminate the effect of the dexamethasone sodium phosphate injection on the following experiments. Furthermore, fecal pellets were collected every day and suspended in 100 μL of 2.5% potassium dichromate solution for the detection of *C. parvum*. The microscopic smear examination found that *C. parvum* oocysts could be detected in the feces on the fifth day after parasitic inoculation.

Before the parasitic infection and decapitation, the mice were weighed and the tissues and organs—including the adrenal gland, spleen, thymus, testis, epididymis, seminal vesicle, and preputial gland—were dissected out. All samples were rinsed in 0.9% normal saline and dried with filter paper. The absolute weights of the samples were recorded, and the mass indexes of these samples were calculated with the following formula: mass index = absolute organ weight (mg) × 100/bodyweight (g).

### 2.7. Extraction of Volatile Pheromones from the Preputial Gland Secretions

After the male mice were sacrificed, the preputial glands were separated and squeezed with tweezers, the extruded light yellow secretion was aspirated with a disposable capillary glass tube and finally transferred to a centrifuge tube. A total of 10 μL of dichloromethane was added to 1 mg of the separated secretions, and the mixture was left to set for at least 12 h on ice. The upper layer was discarded and the lower liquid was stored at −20 °C for chemical analysis until use [24,25].

### 2.8. Urine Collection

Two weeks after parasitic infection, the urethra and anus of the mice were cleaned with 75% ethanol and the male mice were transferred to a clean cage; the cage was covered with a wire mesh (0.5 × 0.5 cm) about 2 cm from the bottom. After the mice urinated, the urine was aspirated with a disposable capillary glass tube and transferred to a centrifuge tube. It should be noted that when mice urinated, the urine contaminated by feces was discarded in the urine collection procedure. The device used for urine collection is presented in Supplementary File S1. The urine was collected to total at least 1 mL per day for a single mouse and stored at −20 °C immediately after collection. Urine collection was performed for two days at 9:00 to 16:00, and the duration of a single collection for any mouse did not exceed 30 min. Equal amounts of the urine collected from the same mouse over two days were mixed and stored at −20 °C for testing.

### 2.9. Behavioral Tests

The preference of each female mouse to male mouse urine was tested as described previously [26,27]. A female mouse was transferred to the test room under dim light before each trial, 2 μL of urine was presented to the mouse with a disposable glass (i.d. 1.1–1.2 mm, o.d. 1.3–1.4 mm, 15 cm length) capillary through the cage cover, and one end of the capillary was sealed with plasticine. The liquid bottom was about 1 cm away from the capillary tip, so that the mice could not come into direct contact with the sample, and only volatiles were accessible to the subjects. Two different types of urine samples (infection group and control group) were simultaneously presented to a female mouse: the two capillaries were lowered through the wire lid and kept approximately 2 cm apart. The device used for the behavioral tests is presented in Supplementary File S1. The attractiveness of the male mouse urine was measured by recording the cumulative duration of the behavior of female mice sniffing urine samples within 3 min. Each mouse was chosen randomly and used only once per day, and any female mouse that did not respond to the two capillaries over the first 3 min was excluded for the day. A total of 24 ICR/CD-1 female mice were used in the behavioral tests for three consecutive days, and the urine samples collected from the infection and control groups were all tested.

### 2.10. Determination of Serum Corticosterone and Testosterone

Mice were placed under anesthesia with diethyl ether and were bled from the retro-orbital sinus into tubes. Blood samples were left at 37 °C for 1 h, and the serum was separated by centrifugation at 4000× *g* rpm for 10 min at 4 °C. The serum was frozen and stored at −80 °C until use. The concentrations of serum corticosterone and testosterone were determined by radioimmunoassay (RIA) at the Beijing Sino-UK Institute of Biological Technology.

### 2.11. Quantitative Real-Time RT-PCR (qRT-PCR)

Mice were sacrificed by neck displacement, and the livers were separated immediately and frozen in liquid nitrogen. Total RNA was extracted from 50–100 mg of the liver tissue with TRIzol reagent (Invitrogen, Berkeley, CA, USA). The purity of RNA was assessed from the ratio of optical densities at 260 nm and 280 nm, and the integrity was checked by 1% agarose gel electrophoresis. The first-stranded cDNA was synthesized with 2 µg of the total RNA by PrimeScript™ II First Strand cDNA Synthesis Kit (TaKaRa, Beijing, China). Afterwards, the mRNA abundances of MUP variants were measured with the TaKaRa SYBR Green QPCR kit (TaKaRa, Beijing, China) performed on an ABI Prism 7000 sequence detection system. The primer sequences used in qRT-PCR are listed in Table 1, and *β-actin* was used as a normalization control to correct for any loading discrepancies.

### 2.12. SDS-PAGE Assay

SDS-PAGE was performed as previously described [28,29]. For the expression analysis of MUP in urine, 10 µL of mouse urine was mixed with 5 × SDS-PAGE loading buffer [250 mM Tris-HCl at pH 6.8, 10% (*w*/*v*) SDS, 0.5% (*w*/*v*) bromophenol blue, 50% (*v*/*v*) glycerol, and 5% (*v*/*v*) β-mercaptoethanol] and heated at 95 °C for 10 min. Then, it was loaded for 15% SDS-PAGE and stained with Coomassie brilliant blue G-250 (Solarbio, Beijing, China). The Quantity One 1-D software (BioRad, Berkeley, CA, USA) was used to perform densitometric analysis on the band with a protein molecular weight of about 18 kDa.

### 2.13. GC-MS Analysis

Gas chromatography-mass spectrography (GC-MS) was performed on an Agilent Technologies Network 6890 N GC system combined with a 5973 Mass Selective Detector. The GC system was equipped with an HP-5MS capillary column (30 m long × 0.25 mm i.d. × 0.25 μm film thickness). The carrier gas was helium at a flow rate of 1.0 mL/min (purity ≥ 99.999%). The temperature of the injection port was 230 °C, the initial temperature of the furnace temperature was 50 °C, and the temperature was programmed to increase to 150 °C at a rate of 5 °C/min, and then to 230 °C at a rate of 10 °C/min and maintain this temperature for 10 min to clean the column after running the samples. For the mass spectrometer, the electron impact ionization was set to 70 eV, the temperature of the transfer tube was set to 280 °C, and the scanning nucleocytoplasmic ratio (*m*/*z*) was 30–450 amu. The preputial gland secretions (3 μL) were injected manually in a split (10:1) mode. The mass spectra were searched using the NIST/EPA/NIH 2002 mass spectral library, and data processing was performed with Xcalibur software (Version 4.2). Preliminary qualitative analysis of the target compound was carried out by comparing the mass spectrum of the peaks on the gas chromatogram with the mass spectrum library (NIST2002), and referring to the compound that was identified in the published paper [30].

Quantitative comparisons of specific compounds between groups were performed in terms of absolute abundance and relative abundance of the compounds. The absolute abundance of a particular compound was quantified as the chromatographic peak area, and the relative abundance was the percentage of the abundance of a particular compound over the total chromatographic peak area of all identified compounds in the source sample.

## 3. Results

### 3.1. Dexamethasone Sodium Phosphate Treatment Decreased Immunity

**Figure 1:**
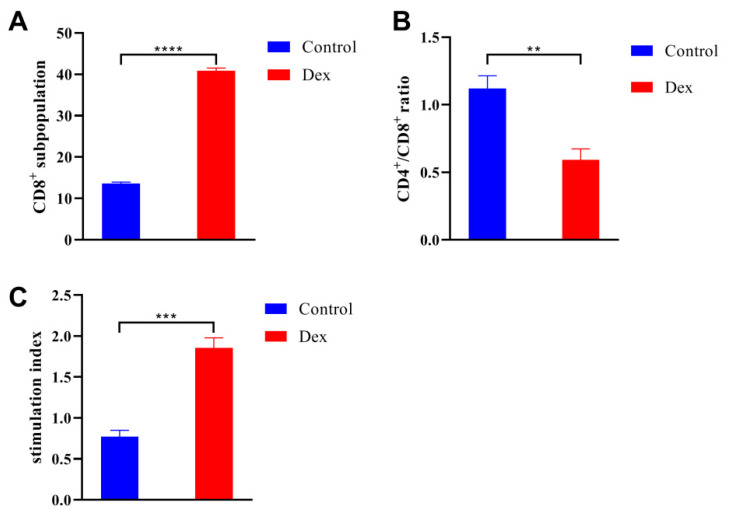
Dexamethasone sodium phosphate treatment lowered the immunity of ICR/CD-1 male mice. (**A**) Dexamethasone sodium phosphate treatment increased the percentage of CD8^+^ cells in the whole white blood cells. (**B**) The CD4^+^/CD8^+^ ratio from the Dex group was significantly lower than that in the control group. (**C**) The dexamethasone sodium phosphate treatment suppressed the viability of splenic T lymphocyte cells. All data are presented as means ± standard deviation. Two-way ANOVA was used for comparison between the two groups, and all statistical analyses were performed with GraphPad Prism 8. **** *p* < 0.0001; *** *p* < 0.001; ** *p* < 0.01.

The CD8^+^ white blood cells could respond to immunodepressant and parasitic infection, and their percentage was increased significantly after dexamethasone sodium phosphate treatment (Figure 1A). The CD4^+^/CD8^+^ ratio was a reflection of the immune system status, and the increased count of CD8^+^ white blood cells resulted in a remarkable reduction of the CD4^+^/CD8^+^ ratio (Figure 1B). Furthermore, the results of lymphocyte proliferation assays indicated that the immunodepressant significantly decreased the T lymphocyte cell viability (Figure 1C). These data showed that the dexamethasone sodium phosphate treatment could lower immunity and facilitated the following parasitic infection.

### 3.2. C. parvum Infection Reduced Physiological Indexes

**Figure 2:**
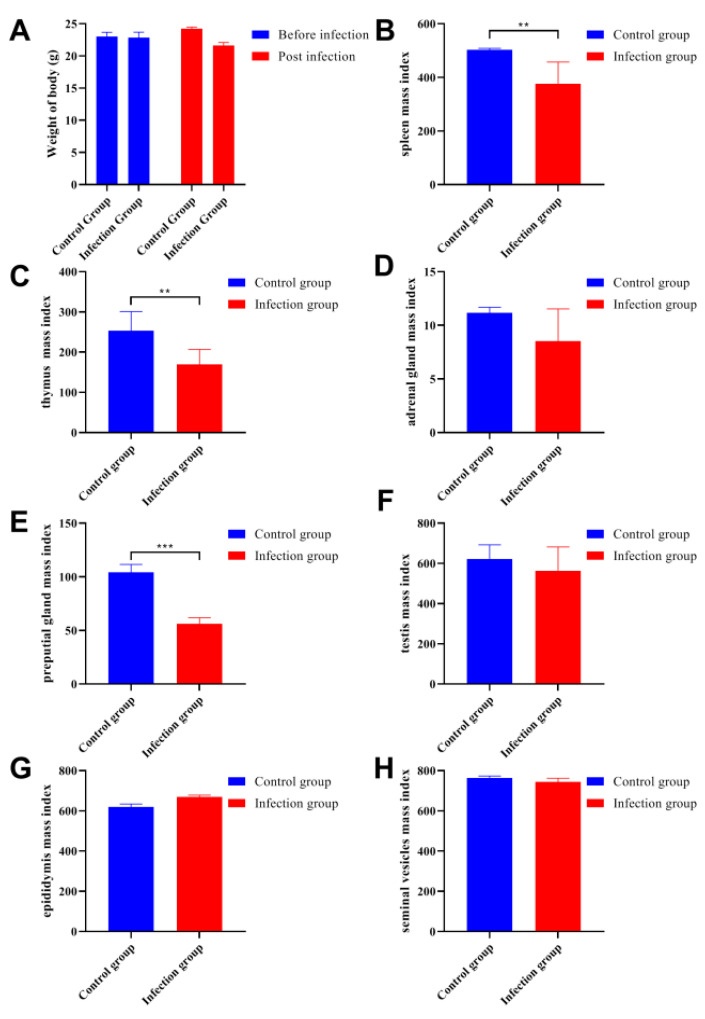
*C. parvum* infection decreased the physiological indexes. (**A**) The comparison of body weight between the infection group and control group before and after parasitic infection. (**B**–**H**) The changes in mass indexes for different organs between the two groups are presented before and after parasitic infection,. The mass indexes of the spleen, thymus, and preputial gland were reduced after parasitic infection. All data are presented as means ± standard deviation. Two-way ANOVA was used for significance analysis, and all statistical analyses were performed with GraphPad Prism 8. *** *p* < 0.001; ** *p* < 0.01.

Before *C. parvum* infection, there was no difference in body weight between the infection group and control group. A total of 14 days after parasitic infection, the bodyweight in infection group was decreased but the difference was not significant under statistical analysis between the two groups (Figure 2A). After parasitic infection, the immune-related organs such as the spleen and thymus had decreased mass indexes (Figure 2B,C). The mass index of adrenal gland between the two groups showed no significant differences (Figure 2D). For the sex organs, only the mass index of the preputial gland from the infection group was decreased and showed significant differences (Figure 2E). Other sex organs including the testis, epididymis, and seminal vesicles did not show obvious differences when compared with the control group (Figure 2F–H). 

### 3.3. Infection with C. parvum Decreased the Attractiveness of Male Mouse Urine to Females

**Figure 3:**
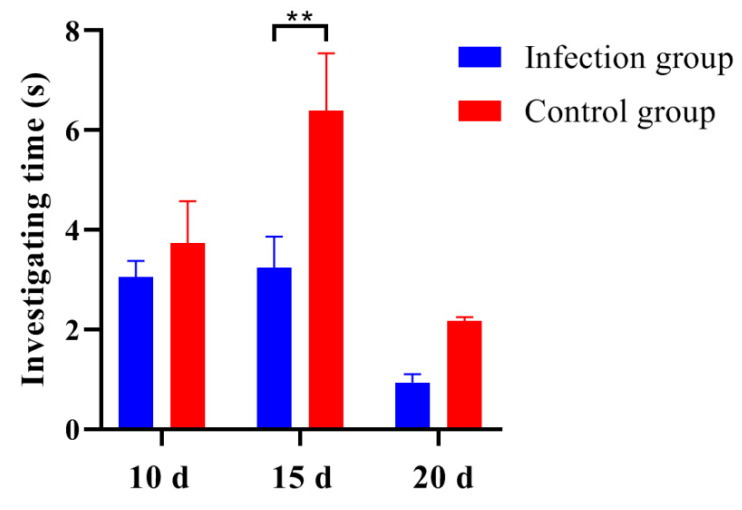
The parasitic infection decreased the attractiveness of ICR/CD-1 male mouse urine to females. Attractiveness was evaluated by recording the cumulative duration of female mice in sniffing and exploring the different urine samples within 3 min. All data are presented as means ± standard deviation. Two-way ANOVA was used for significance analysis, and all statistical analyses were performed with GraphPad Prism 8. ** *p* < 0.01.

Behavioral tests were performed at 10, 15, and 20 days post infection. The results indicated that the female mice spent a longer time sniffing and exploring the male mice urine from the control group, and the data showed significant differences at 15 days post infection (Figure 3). These results indicated that infection with *C. parvum* reduced the urine attractiveness of ICR/CD-1 male mice to females.

### 3.4. C. parvum Infection Reduced the Gene Expression of MUP

**Figure 4:**
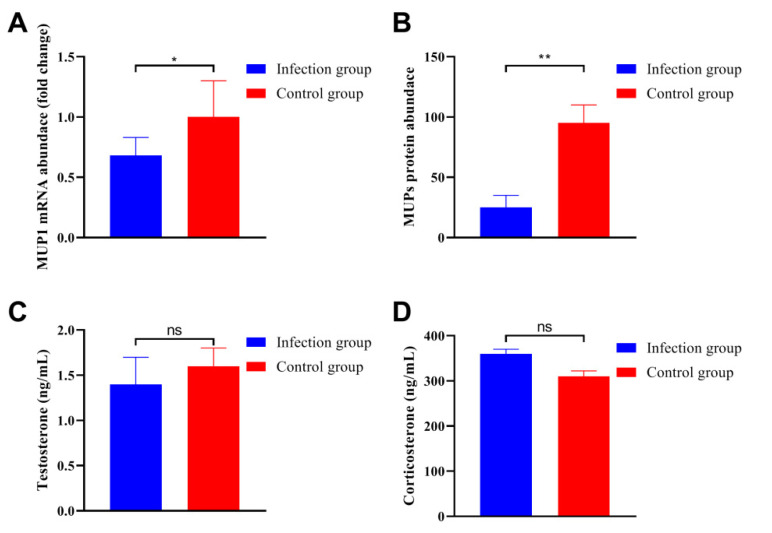
*C. parvum* infection reduced the expression of MUP variants and showed no influence on the hormone level. (**A**) The expression level of MUP variants in the liver tissue was significantly down-regulated after parasitic infection. (**B**) SDS-PAGE analysis indicated that the protein expression level of MUP was significantly decreased for the mice infected with *C. parvum*. The levels of testosterone (**C**) and corticosterone (**D**) in serum showed no significant differences between the infection group and control group. All data are presented as means ± standard deviation. Two-way ANOVA was used for significance analysis, and all statistical analyses were performed with GraphPad Prism 8. * *p* < 0.1; ** *p* < 0.01; ns non-significant.

To determine the gene expression level of MUP in live tissue, the total RNA was extracted and real-time quantitative PCR was performed on the fragment of MUP variants. The results indicated that *C. parvum* infection significantly down-regulated the expression level of MUP variants (Figure 4A). These results were further identified by SDS-PAGE analysis, the band densitometric of MUP in male mice infected with *C. parvum* was significantly lower than that in the control group (Figure 4B). Radioimmunoassay was performed to determine the levels of testosterone and corticosterone in serum. The results indicated that the levels of these two hormones showed no significant differences between the two groups (Figure 4C,D).

### 3.5. C. parvum Infection Significantly Reduced Pheromone Content in the Secretions of Mouse Prepuce Gland

**Figure 5:**
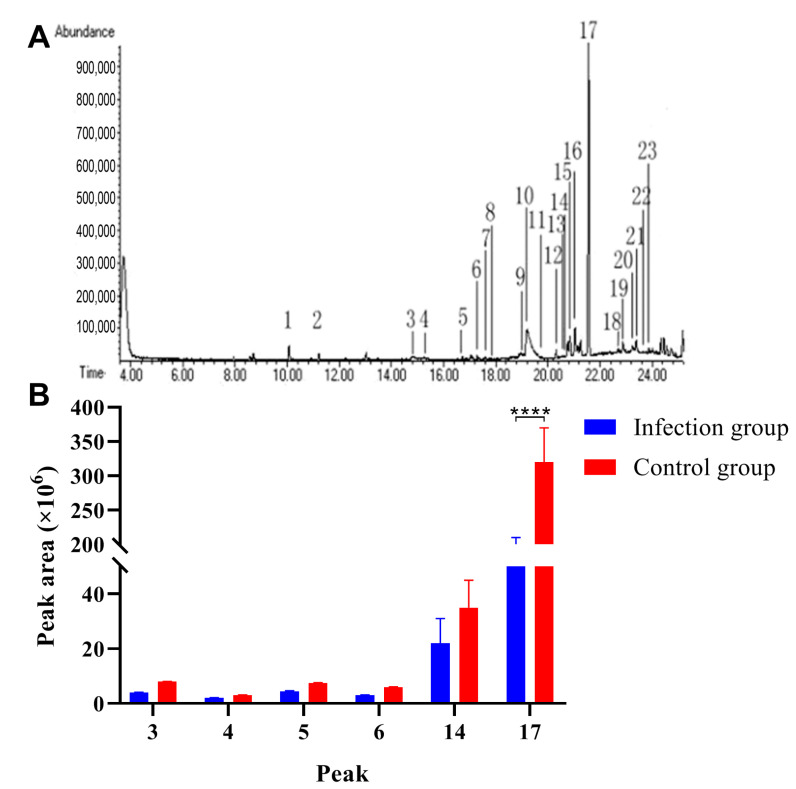
*C. parvum* infection significantly reduced the pheromone contents in the secretions separated from the preputial gland. (**A**) Representative gas chromatogram of the secretions from the infection group. (**B**) The absolute abundances of Z-7-tetradecen-1-ol, 1-tetradecanol, Z-5-tetradecenol acetate, 1-tetradecanol acetate, 1-hexadecanol acetate, and 1-hexadecanol acetate in the secretions of the preputial gland were decreased after parasitic infection. **** *p* < 0.0001.

GC-MS analysis was performed to analyze the volatile compounds in the secretions separated from the preputial gland, and the representative gas chromatogram from the infection group was presented in Figure 5A. The date were compared with the mass spectrometry libraries: published papers were also referenced [31,32]. In brief, we identified a total of 23 volatile compounds in the secretions of the preputial gland. The absolute abundance (peak area) and relative abundance (percentage of total) of the volatile compounds in the two groups are compared and listed in Table 2. The results indicated that the absolute abundances of Z-7-tetradecen-1-ol, 1-tetradecanol, Z-5-tetradecenol acetate, 1-tetradecanol acetate, 1-hexadecanol acetate, and 1-hexadecanol acetate from the infection group were significantly lower than those in the control group (Figure 5B).

## 4. Discussion

For small rodents, the signals produced by one individual and picked up by another play an important role in controlling social behavior [33]. The urine is a rich source of biological signals for male mice [34], which can provide abundant information regarding age, sex, health status, hormonal levels, and genetic background to other individuals [35,36]. For reproduction, female mice are particularly sensitive to the social status of individual males under semi-natural conditions, and only mate with dominant males that are able to successfully defend territories [37]. In brief, the pheromones produced by male mice can provide reliable individual information and have a profound impact on social behavior.

Studies have shown that parasitic infections could lead to reproductive damage, and the main reason for this damage was the lower quality of sperm caused by parasitic infections [38,39,40,41,42]. On the other hand, pathogen infection can reduce the attractiveness of urine from infected male mice [43], and this unhealthy status can even stimulate aversion in females [44]. Although the chemical changes in urine associated with infection are not yet known, mice are able to recognize the subclinical infection or the activation of immune responses [43]. Compared with male mice infected with parasites [45] or worms [46], female mice are more likely to be attracted by the urine of uninfected male mice. Consistent with previous studies, our data revealed that *C. parvum* infection reduced the urine attractiveness of the male mice to female mice; furthermore, the pheromone contents in the preputial gland were also reduced in the infected male mice.

The preputial gland is a large lipid and hormone secreting sebaceous organ of mice [47]. In this study, we analyzed the change in pheromone contents in the preputial gland through GC-MS after parasitic infections. The results indicated that the absolute abundances of Z-7-tetradecen-1-ol, 1-tetradecanol, Z-5-tetradecenol acetate, 1-tetradecanol acetate, 1-hexadecanol acetate, and 1-hexadecanol acetate from the infection group were significantly lower than those in the control group. Other studies have reported that hexadecanol and hexadecyl acetate were the major components of preputial gland secretion, and the addition of these two components could recover the urine attractiveness to females of urine produced by castrated male mice [48]. Our results indicated that parasitic infection decreased the pheromone contents in preputial gland secretions and further reduced the urine attractiveness to female mice.

In this study, ICR/CD-1 male mice were used to explore the relationship between parasitic infection and urine attractiveness. It should be noted that by preferentially mating with healthy males, females were less likely to be infected with parasites, and they could pass on good genes to their offspring from attractive males [49]. ICR/CD-1 mice are a *Foxn1* gene knockout mouse model: this mouse is athymic and T-cell deficient, which makes the mouse immunodeficient and easily susceptible to parasitic infection [50]. Furthermore, ICR/CD-1 mice have also been used for reproductive research in other studies [51,52,53].

MUP is the transporter of a variety of ligands, including pheromones, steroid hormones, retinoids, and lipids [54], and different MUP subtypes are highly homologous [55]. MUP is mainly expressed in liver with a sex-dimorphic pattern, and the expression levels are several times higher in males than in females [54]. The expression level of MUP can be regulated by multiple hormones including testosterone, growth hormone, and thyroxine [4]. In this study, parasitic infection reduced the expression level of MUP, but the levels of testosterone and corticosterone in serum were not affected significantly, which indicated that parasitic infection could reduce the MUP expression level directly.

In summary, we report that *C. parvum* infection could suppress the synthesis of MUP: both the mRNA and protein abundance were down-regulated after parasitic infection and these results enriched the regulation and control methods of MUP production. Furthermore, some studies have reported that parasitic infection could lead to reproductive injury by reducing the sperm quality; in this study, we found that parasitic infection could down-regulated the pheromone contents in urine and this phenomenon could also affect the reproductive capacity.

## 5. Conclusions

This study revealed that the urine attractiveness of ICR/CD-1 male mice was decreased after parasitic infection when compared with the uninfected group. Both the gene expression level and protein concentration of MUP in liver and urine were down-regulated after parasitic infection. The results of GC-MS analysis indicated that the contents of different pheromones were significantly reduced after parasitic infection. In summary, this study revealed that infection with *C. parvum* affects the secondary sexual characteristics of male ICR/CD-1 mice and alters the pheromone contents in the foreskin gland.

## Figures and Tables

**Table 1:** Primers used in this study.

Gene Name	Sequence (5′-3′)	Length (bp)
*MUP*	5′-GTGAGAAGCATGGAATCCTTAGAGA-3′	103
	5′-TCAACACTGGAGGCTCAGGC-3′	
*β-actin*	5′-AGCCTTCCTTCTTGGGTATGG-3′	104
	5′-TGTGTTGGCATAGAGGTCTTTACG-3′	

**Table 2:** Comparison of the absolute content (peak area) and relative amount (% of total) of the volatile compounds in the preputial gland between the two groups.

Peak	Compound Name	Peak Area		% of Total	
		Control group	Infection group	Control group	Infection group
1	E-β-farnesene	3.23 × 10^7^ ± 3.54 × 10^6^	2.9 × 10^7^ ± 2.17 × 10^6^	5.48 ± 0.29	6.44 ± 0.41
2	2-Heptanone	1.92 × 10^7^ ± 2.09 × 10^6^	1.44 × 10^7^ ± 1.27 × 10^6^	3.27 ± 0.18	3.72 ± 0.24
3	Z-7-tetradecen-1-ol	7.76 × 10^6^ ± 7.34 × 10^5^	4.38 × 10^6^ ± 5.06 × 10^5^	1.36 ± 0.10	1.17 ± 0.16
4	1-Tetradecanol	2.97 × 10^6^ ± 3.30 × 10^5^	1.95 × 10^6^ ± 2.12 × 10^5^	0.51 ± 0.04	0.51 ± 0.06
5	Dimethyl sulfone	7.24 × 10^6^ ± 7.14 × 10^5^	3.57 × 10^6^ ± 2.97 × 10^5^	1.27 ± 0.11	0.94 ± 0.08
6	6-Hydroxy-6-methyl-3-heptanone	5.34 × 10^6^ ± 5.41 × 10^5^	2.91 × 10^6^ ± 3.03 × 10^5^	0.92 ± 0.06	0.73 ± 0.03
7	R,R-3,4-dehydro-exo-brevicomin	1.24 × 10^6^ ± 1.59 × 10^5^	1.05 × 10^6^ ± 1.97 × 10^5^	0.21 ± 0.02	0.25 ± 0.04
8	(S)-2-sec-butyl-4,5-dihydrothiazole	5.60 × 10^5^ ± 4.90 × 10^4^	3.99 × 10^5^ ± 9.37 × 10^4^	0.10 ± 0.01	0.09 ± 0.02
9	Z-9-Hexadecenol	9.29 × 10^6^ ± 1.21 × 10^6^	6.86 × 10^6^ ± 8.09 × 10^5^	1.54 ± 0.09	1.73 ± 0.12
10	1-Hexadecanol	1.07 × 10^8^ ± 1.28 × 10^7^	8.25 × 10^7^ ± 9.34 × 10^6^	17.85 ± 0.80	20.84 ± 1.24
11	1-Pentadecanol acetate	1.81 × 10^6^ ± 2.31 × 10^5^	1.45 × 10^6^ ± 1.84 × 10^5^	0.30 ± 0.02	0.35 ± 0.01
12	Z-9-Hexadecenol acetate	3.88 × 10^6^ ± 5.00 × 10^5^	2.75 × 10^6^ ± 3.03 × 10^5^	0.64 ± 0.01	0.68 ± 0.02
13	1-Heptadecanol	1.02 × 10^6^ ± 1.24 × 10^5^	9.47 × 10^5^ ± 1.03 × 10^5^	0.18 ± 0.02	0.25 ± 0.03
14	1-Hexadecanol acetate	3.79 × 10^7^ ± 5.01 × 10^6^	2.37 × 10^7^ ± 3.11 × 10^6^	6.24 ± 0.20	5.68 ± 0.18
15	Isomer of Z-11-Hexadecanol acetate	1.26 × 10^7^ ± 1.88 × 10^6^	8.83 × 10^6^ ± 1.50 × 10^6^	2.10 ± 0.18	2.06 ± 0.14
16	Z-11-Hexadecenol acetate	2.71 × 10^6^ ± 7.26 × 10^5^	2.25 × 10^6^ ± 4.96 × 10^5^	0.59 ± 0.25	0.63 ± 0.15
17	1-Hexadecanol acetate	3.14 × 10^8^ ± 4.37 × 10^7^	1.99 × 10^8^ ± 3.09 × 10^7^	50.96 ± 1.25	46.26 ± 2.23
18	1-Heptadecanol acetate	2.38 × 10^6^ ± 3.30 × 10^5^	2.28 × 10^6^ ± 2.46 × 10^5^	0.39 ± 0.02	0.57 ± 0.03
19	1-Heptadecanol acetate	4.30 × 10^6^ ± 5.43 × 10^5^	3.66 × 10^6^ ± 4.16 × 10^5^	0.71 ± 0.02	0.91 ± 0.02
20	1-Octadecanol	6.11 × 10^6^ ± 7.81 × 10^5^	5.52 × 10^6^ ± 6.21 × 10^5^	1.01 ± 0.05	1.41 ± 0.10
21	1-Heptadecanol acetate	4.68 × 10^6^ ± 5.83 × 10^5^	3.75 × 10^6^ ± 5.25 × 10^5^	0.77 ± 0.02	0.89 ± 0.02
22	Z-7-Octadecenol acetate	5.06 × 10^6^ ± 7.17 × 10^5^	3.91 × 10^6^ ± 4.99 × 10^5^	0.82 ± 0.03	0.98 ± 0.08
23	Octadecanol acetate	1.68 × 10^7^ ± 2.08 × 10^6^	1.23 × 10^7^ ± 1.87 × 10^6^	2.77 ± 0.08	2.92 ± 0.12

## Data Availability

The original data presented in the study are included in the article and Supplementary Materials. Further inquiries can be directed to the corresponding author.

## Supplementary Materials

The following supporting information can be downloaded at: www.mdpi.com/10.3390/ani13040756/s1, Supplementary File S1 is the schematic diagram of the device used for urine collection and behavioral tests.
