# Supplementary material for: Infection with Cryptosporidium parvum Affects Secondary Sexual Characteristics of Male Mice by Altering the Pheromone Content in Preputial Gland
Source: Animals (Basel). 2023 Feb 19;13(4):756. doi: 10.3390/ani13040756 (PMC9952591; doi:10.3390/ani13040756)
Supplement: Supplementary file 1 [file animals-13-00756-s001.zip › animals-2185704-supplementary.pdf]

Supplementary File S1

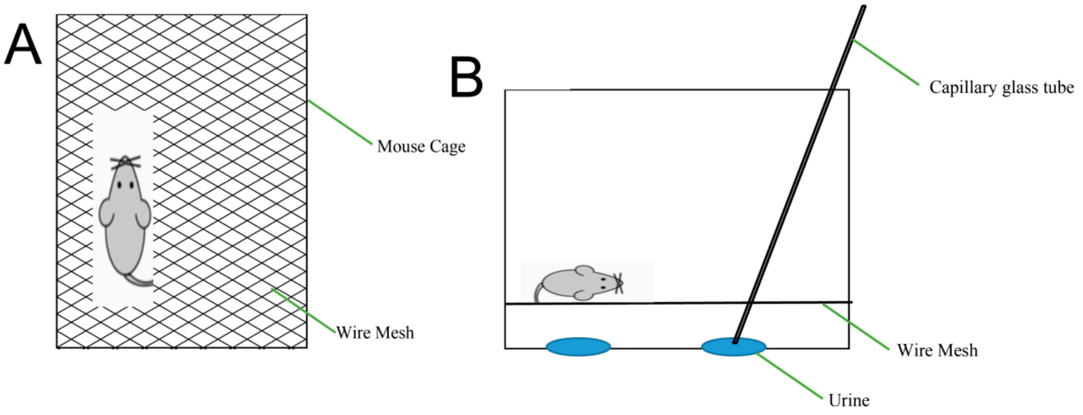

Figure S1 Schematic diagram of the device used for urine collection. (**A**) Mouse was placed in a clean cage, the cage was covered with a wire mesh (0.5 × 0.5 cm) about 2 cm from the bottom. (**B**) After the mice urinated, the urine was aspirated with a disposable capillary glass tube and transferred to a centrifuge tube. When mice urinated, the urine contaminated by feces was discarded in the urine collection procedure.

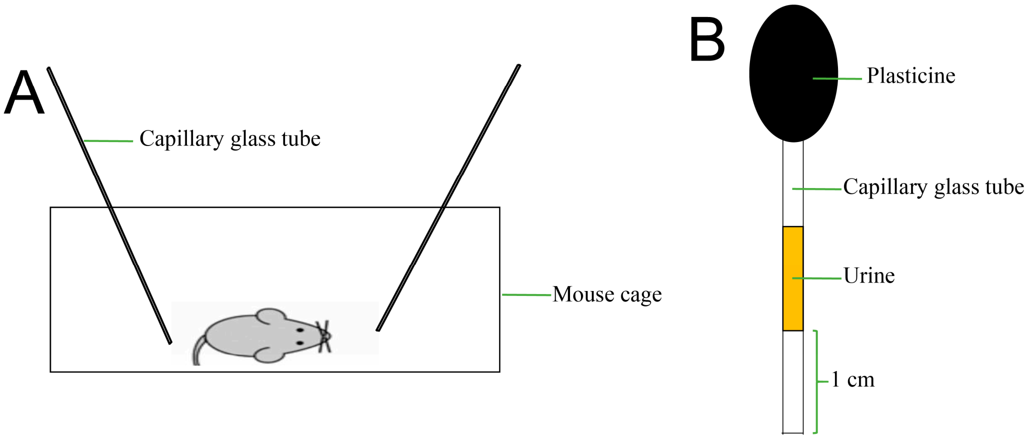

Figure S2 Schematic diagram of the device used for behavioral test. (**A**) In the behavioral test, female mice was placed in a clean cage, capillary glass tubes with urine samples from infection group and control group were presented to the mouse through the cage cover. (**B**) The capillary glass tube carried 2 μL of urine, and one end of the capillary was sealed with plasticine. The liquid bottom was about 1 cm away from the capillary tip.
